# Role of family functioning and health-related quality of life in pre-school children with dental caries: a cross-sectional study

**DOI:** 10.1186/s12955-021-01828-3

**Published:** 2021-08-03

**Authors:** Sobia Bilal, Anshad Mohamed Abdulla, Niekla Survia Andiesta, Muneer Gohar Babar, Allan Pau

**Affiliations:** 1 Division of Children and Community Oral Health, School of Dentistry, International Medical University, Kuala Lumpur, Malaysia; 2 Department of Pediatric Dentistry and Orthodontics, College of Dentistry, King Khalid University, Abha, Kingdom of Saudi Arabia

**Keywords:** Dental caries, Health-related quality of life, Pre-school children, Family functioning, Impact

## Abstract

**Background:**

The aim of this cross-sectional study was to evaluate the effect of family functioning on oral health related quality of life (OHRQoL) and dental caries status among 4- to 6-year-old Chinese pre-school children in Malaysia.

**Methodology:**

This study was approved by the institutional Joint Research and Ethics Committee, International Medical University, Malaysia (number 373/2016); consisted of 180 eligible pre-school children from a private school. Study tools included demographic, clinical oral health data form, the Early Childhood Oral Health Impact Scale (ECOHIS) and family functioning—12-item general functioning subscale. Written consent was sought prior to data collection. Data were analysed by SPSS v.22.0; descriptive statistics for socio-demographic details, clinical information, HRQoL and FAD scores. The parametric tests included independent sample t test and ANOVA to evaluate the associations between the dependent variable. Binary logistic regression models were applied to assess the impacts on OHRQoL (*P* value < 0.05).

**Results:**

A response rate of 80.5% was achieved. Sample means for FAD-GF12 scores ranged < 2, indicating normal family functioning. The ECOHIS scores mean was 0.52 (SD = 0.68). In the children impact section the highest score of impacts 20 was noted followed by family impact section with the score of 12. It was observed that children with dental caries had a significant (*P* = 0.014) negative impact on OHRQoL (mean 0.72, SD = 0.50) as compared to children with no caries (mean 0.34 SD = 0.39). The bivariate regression analysis after getting adjusted indicated three predictors associated to poor HRQoL: having two or more siblings (OR = 4.73, *P* = 0.013), relationship (mother) of the respondent to the child (OR = 2.86, *P* = 0.013) and having dental caries (OR = 8.12, *P* ≤ 0.001).

**Conclusion:**

Findings of this study indicates that number of siblings, relationship of the respondent to the child and dental caries status are independently associated with health-related quality of life. However, family functioning does not mediate poor HRQoL in association to dental caries.

## Background

Dental caries is a multifactorial disease, and occurs due to complicated interaction between the carcinogenic microbial flora, high risk behaviours and social influences [1, 2]. Children in the age group of 1–5 years have a caries pattern that differs from that of adults, It is termed as “Early Childhood Caries (ECC)” when describing any form of caries in infants and preschool children [3]. In a systematic review almost 90 risk factors for ECC were described [4], due to this complex aetiology of ECC, researchers are now focussing into other risk factors involved besides inappropriate feeding and oral hygiene practices [5, 6]. The scope that dental caries in young children poses as a public health problem warrants further investigation into the natural progression of this disease. One such factor that could mediate the development of ECC is family functioning [7]. The factor of family functioning may also affect child health problems e.g. dental caries. For the basic needs such as food, shelter and security; pre-school children are entirely reliant on their families. Preferably these families should strive towards a conducive environment that favours the overall well-being of its individual members [8]. Parental role is crucial in manifesting particular behaviours related to ECC, specifically children's oral hygiene practices and frequency of sugar consumption [9]. Along with individual parental role, good family interaction is vital to create a healthy environment that reflects on child’s emotional and physical health [10]. Family functioning is therefore an important family characteristic to consider when examining the child’s caries risk.

Untreated decay can cause pain and impair the nutritional status and physical growth. ECC is a public health problem in Malaysia affecting 75% of Malaysian preschool children, most of which remains untreated [11]. Based on the report by Health Information Management System, Malaysia in 2012, referrals of approximately 4000 children to hospital paediatric dental services were made due to ECC [12]. Studies have shown that dental caries present negative impact on quality of life of children and their families. The oral health-related quality of life (OHRQoL) is a subjective and multidisciplinary aspect which has been studied in several countries. It embraces broad issues in its concept, such as physical, family and leisure characteristics, etc. [13–16] The theoretical model explained by Kramer et al. in a study done among Brazilian pre-school children, showed association of sociodemographic, oral diseases and OHRQoL [15].

Good family functioning and strong family relationships are important predictors for children’s overall well-being which influences on their physical, social and emotional development [17]. The evidence behind the association between family functioning and HRQoL is not considerable. Related to dental caries, family functioning can be considered as a modifiable risk factor; it can be a protective factor under good family functioning and on the other hand as a risk factor under poor family functioning. Much is understood about dental caries aetiology and the individual characteristics that are associated with it [2, 4, 5]. Less well understood is the effect of family functioning of children, especially in the context of diverse multi-ethnic family relationships within urban and rural settings, and rapid urbanisation. Family functioning is reported to be linked with child development process including physical and mental well-being [12, 14, 18]. Additionally, this supportive and organized environment also reflects on children’s good oral health behaviours ^7^. Also previous studies explored the social and psychosocial factors on child oral health and found that the relationships were not always independent of socioeconomic factors [19–22]Malaysia is a multi-ethnic country with the population of 28million. Majority of the population belongs to indigenous group called Bumiputras or Malays (nearly 70%), followed by Chinese (23%) and Indians (7%) ethnic groups [23]. In this study only Chinese pre-school children where included as we believe the concept of family functioning might vary in different ethnic population and in future we would like to explore this concept in the remaining two ethnic groups. The aim of this cross-sectional study was to evaluate the effect of family functioning and caries status on oral health related quality of life (OHRQoL) in a sample of 4 to 6-year-old Chinese pre-school children in Malaysia. The objectives were (1) to investigate the association between sociodemographic characteristics and family functioning (2) to investigate the association between sociodemographic characteristics, family functioning and dental caries status. (3) to investigate the association between sociodemographic characteristics, family functioning, dental caries status and HRQoL. (4) to explore whether family functioning could mediate the relationship between childhood dental caries and OHRQoL (5) to explore the predictors of HRQoL among the study sample.

## Materials and methods

This cross-sectional study was approved by the institutional Joint Research and Ethics Committee (number 373/2016). The sample consisted of 180 eligible pre-school children from a private school in Malaysia. Limited oral health data is available on private schools as compared to public schools in Malaysia. Bases on convenience sampling, the school selected was a private setting with pre-school, primary and secondary sections. The children studying in this school learn Mandarin as their first language. The study sample included 4–6 years old Chinese pre-school children. There were 7 classes with around 25–26 students in each class. All the students were included in the study regardless of the gender. Children who had reached the age of six but have not had their seventh birthday on the date of examination were included in the study.

### Data collection measures

The selected questionnaires were translated into Mandarin to address the language barrier. Content and face validity were ensured by getting the translated versions checked by 10 Mandarin speaking lay people. The following forms and questionnaires were used for data collection:

1. Demographic questionnaire: A demographic questionnaire assessing the family characteristics: age of the child, gender (male/female), staying with (both parents/single parent), number of siblings (no sibling/one sibling/two or more), relationship to child (father/mother), household income (up to Malaysian Ringgit (RM) 5000/more than RM5000) was completed by parents. Based on the income classification by household, Malaysians are categorised into three different income groups: Top 20% (T20) with income more than RM10,000, Middle 40% (M40) with income range between RM5000–RM10,000, and Bottom 40% (B40) with income below RM 5000. [24]
2. Clinical Oral Health data form: Clinical oral health data were collected through this form. The number of decayed, missing (due to caries) and filled deciduous teeth (dmft) were recorded. The diagnosis of caries was based on the dmft index, [25] which allows professionals to measure and to compare the dental caries experience in populations.
3. Oral health-related quality of life (OHRQOL) assessed using the ECOHIS (Early Childhood Oral Health Impact Scale): Pahel et al. [26] developed this measure which was later validated for various populations. The Malaysian validated version by Hashim et al. [27] was used in this study. This 13-item measure was developed to evaluate parents perception on OHRQoL of preschool children and comprise two sections: the Child Impact Section (CIS) with four domains (child symptoms, function, psychological and self-image/social interaction); and the Family Impact Section (FIS) with two domains (parent distress and family function). Likert scale was used to capture the responses with 0 = never; 1 = hardly ever; 2 = occasionally; 3 = often; 4 = very often; 5 = don’t know.
4. Family functioning—12-item general functioning subscale (GF-12): the Family Assessment Device (FAD) is a 60-item self-report questionnaire developed to measure family functioning and the GF-12 is its 12-item subscale [28]. This measure was assessed among different populations and was proved to be free from cultural bias [29] with good internal consistency. The use of this subscale is recommended as a standalone to measure the overall family functioning due to high correlation with other FAD scales [30]. This subscale was translated in Mandarin (Chinese version of the GF 12 subscale) [31] The cut-off score is 2, a score of 2.00 or above indicates problematic family functioning [28].

### Data collection process

Families of children eligible for inclusion were approached and invited to participate. An information leaflet, consent form and questionnaires on sociodemographic, ECOHIS and FAD were given to the children to take home to their parents/caregivers via their homework diaries. The primary carer was instructed to provide written consent to participate in the study and complete the questionnaires. The filled questionnaires were collected back from the school children by the class teachers and were handed over to the research team. The data collection team included 15 dental undergraduate students and six dentists. The team was standardized and calibrated through half a day training workshop by Cariology team at IMU. This was conducted to ensure the reliability of the measurements in an oral examination. After obtaining written consent, a one-day visit was arranged to the selected school where oral examinations were carried out by the standardized team.A visual, oral examination of the participating children was carried out using disposable mouth mirrors, without invasive probing, and was diagnosed at the cavitation stage. The children were examined while sitting on a chair under natural daylight. Based on WHO recommendation, the teeth examination was started from upper right to upper left and lower left to lower right quadrant and a tooth was considered present when any part of it was visible [32]. Children who were assessed with obvious caries were referred for dental treatment. No radiographs were taken, and no treatment was provided. Translators (schoolteachers or supporting staff) assistance was taken in case of language barrier.

### Data analysis

Data were entered using the software Statistical Package for the Social Science (SPSS), v.22.0 for Windows. Data were analysed using descriptive statistics (frequency, mean and standard deviation) for socio-demographic details, clinical information, HRQoL and FAD scores. For the FAD-GF12 scoring, 4-point Likert scale was used (1 = strongly agree to 4 = strongly disagree). For negative items (1, 3, 5, 7, 9, 11), the scores were reversed and the scores of the 12 items were summed up to generate the total score. The total score is later divided by the number of items on the subscale, this provides a final score ranging from 1.0 indicating best functioning to 4.0 indicating worse functioning. The variable was dichotomised based on the cut-off score set at 2, a score of 2.00 or above indicates problematic family functioning [28].

The total score for ECOHIS was calculated by simply summing up the response codes of the 13 items and all ‘don’t know’ responses were recoded as missing. Thus, the overall score ranged between 0 and 52 (0–36 for the CIS and 0–16 for the FIS). A score for the missing item was assigned based on the average of other responses. This was done in case of two missing responses in CIS or one in FIS. The higher scores indicated enhanced oral health concerns and poorer OHRQoL [15].

The parametric tests were performed to evaluate the associations between the dependent variable (OHRQOL, measured through ECOHIS and family functioning, measured through the GF-12 of the Family Assessment Device) and the independent variable of with or without caries experience. In the comparison of the ECOHIS and GF-12 mean scores, between the groups, the Independent Samples t Test or Analysis of variance (ANOVA) tests were applied.

ECOHIS scores were dichotomized using the median values as a cut-off point [33].

Descriptive, unadjusted analysis was carried out to assess the association of the overall ECOHIS scores with socio-demographics, dental caries status and family functioning. Chi-squared tests, unadjusted and adjusted binary logistic regression models were used to assess the impacts on OHRQoL. All the independent variables including sociodemographic, FAD and dental caries status, repoting a P value 0.2 in the adjusted calculation of covariates were retained in the final regression model [34].

## Results

### Demographic characteristics

The sample comprised of Chinese children (100%) with a response rate of 80.5%. The reason for losses were due to failure to obtain written consent and incomplete responses. The mean age of the children was 5 years and almost all of them (97.2%) were staying with both their parents and half of the total sample (56.3%) had one sibling. The Majority of the questionnaires were responded by mothers (64.6%). Majority of the parents had monthly income of more than RM5000 (78.5%) (Table 1).

**Table 1:** Socio-demographic characteristics of participants (*n* = 144)

Variables	*N* (%)
Total	144 (100)
Age	
4 years	63 (43.8)
5 years	39 (27.1)
6 years	42 (29.1)
Gender	
Male	63 (43.7)
Female	81 (56.3)
Siblings	
No siblings	26 (18.1)
One sibling	81 (56.3)
Two or more	37 (25.7)
Relationship to child	
Father	51 (35.4)
Mother	93 (64.6)
Monthly income	
Up to RM 5000	31 (21.5)
More than RM5000	113 (78.5)

### Family functioning

The mean score for FAD-GF12 was 1.19 (SD 0.392). Significant association was related to parent’s relationship to the child with family functioning (*P* = 0.042) (Table 2). Respondents who reported their relationship to the child as fathers scored lower (1.10) in the FAD-GF12 when compared to mothers (1.24).

**Table 2:** Association between socio-demographic characteristics and GF12 FAD score of the study sample

Variables	FAD mean (SD)	Good FAD *n* (%)	Poor FAD *n* (%)	Total *n* (%)	*P* value
Total	1.18 (0.38)	117 (81.2)	27 (18.8)	144 (100)	
Age					
4 years	1.17 (0.38)	52 (82.5)	11 (17.5)	63 (100)	0.929
5 years	1.21 (0.40)	31 (79.5)	8 (20.5)	39 (100)	
6 years	1.19 (0.39)	34 (81.0)	8 (19.0)	42 (100)	
Gender					
Male	1.16 (0.36)	53 (84.1)	10 (15.9)	63 (100)	0.439
Female	1.21 (0.41)	64 (79.0)	17 (21.0)	81 (100)	
Siblings					
No siblings	1.23 (0.43)	20 (77.0)	6 (23.0)	26 (100)	0.352
One sibling	1.21 (0.41)	64 (79.0)	17 (21.0)	81 (100)	
Two or more	1.11 (0.31)	14 (37.8)	23 (62.2)	37 (100)	
Relationship to child					
Father	1.10 (0.30)	46 (90.2)	5 (9.8)	51 (100)	0.042
Mother	1.24 (0.42)	71 (76.3)	22 (23.7)	93 (100)	
Monthly income					
Lower than RM 5000	1.19 (0.40)	25 (80.6)	6 (19.4)	31 (100)	0.923
RM 5000 and above	1.19 (0.39)	92 (81.4)	21 (18.6)	113 (100)	

### Caries status

The association of sociodemographic and caries status of the sample is presented in Table 3. Statistical significance was noted with number of siblings (*P* = 0.049) and the mean FAD-GF12 Score (*P*  ≤ 0.001).

**Table 3:** Association between socio-demographic characteristics and caries status of the study sample

Socio-demographic	Caries free *n* (%)	Caries *n* (%)	Total *n* (%)	*P* value
Total	73 (50.7)	71 (49.3)	144 (100)	
Age				
4 years	32 (50.8)	31 (49.2)	63 (100)	0.993
5 years	20 (51.3)	19 (48.7)	39 (100)	
6 years	21 (50.0)	21 (50.0)	42 (100)	
Gender				
Male	32 (50.8)	31 (49.2)	63 (100)	0.983
Female	41 (50.6)	40 (49.4)	81 (100)	
Siblings				
No siblings	13 (50.0)	13 (50.0)	26 (100)	0.050
One sibling	35 (43.2)	46 (56.8)	81 (100)	
Two or more	25 (67.6)	12 (44.4)	37 (100)	
Relationship to child				
Father	28 (54.9)	23 (45.1)	51 (100)	0.455
Mother	45 (48.4)	48 (51.6)	93 (100)	
Monthly income				
Up to RM 5000	17 (54.8)	14 (45.2)	31 (100)	0.602
More than RM5000	56 (49.6)	57 (50.4)	113 (100)	
Family functioning: GF 12				
Good family functioning	72 (61.5)	45 (38.5)	117 (100)	< 0.001
Poor family functioning	1 (3.7)	26 (96.3)	27 (100)	

### HRQoL

The ECOHIS questionnaire were answered by parents, and scores ranged from 0 to 27 with a 0.52 mean (SD = 0.68). In the CIS the highest score of impacts 20 was noted followed by FIS with the score of 12. The overall mean score indicated that dental caries had an adverse effect on OHRQoL (*P* = 0.014). The difference in scores was significant between the levels of dental caries involving all domains in both the CIS and FIS (*P* ≤ 0.001).

### Independent variables with HRQoL

The association of socio-demographic, family functioning and dental caries status characteristics by the ECOHIS scores of the sample are presented in Table 4. ECOHIS scores were dichotomized (ECOHIS ≤ 6 and ECOHIS > 6) using the median values as a cut-off point. The univariate analysis showed that the following variables were statistically significant: relationship to the child (*P* = 0.010), FAD-GF12 (*P* = 0.016) and dental caries status (*P* =  < 0.001).

**Table 4:** The sociodemographic, family functioning and dental caries status characteristics by the ECOHIS scores (total  ≤ 6 and > 6)

Variables	ECOHIS Scores mean (SD)	ECOHIS ≤ 6 *n* (%)	ECOHIS > 6 *n* (%)	Total *n* (%)	*P* value
Total	1.72 (0.44)	78 (54.2)	66 (45.8)	144 (100)	
Age					
4 years	0.45 (0.47)	37 (58.7)	26 (41.3)	63 (100)	0.462
5 years	0.65 (0.51)	18 (46.2)	21 (53.8)	39 (100)	
6 years	0.53 (0.50)	23 (54.8)	19 (45.2)	42 (100)	
Gender					
Male	0.55 (0.51)	32 (50.8)	31 (49.2)	63 (100)	0.474
Female	0.51 (0.48)	46 (56.8)	35 (43.2)	81 (100)	
Siblings					
No siblings	0.47 (0.42)	16 (61.5)	10 (38.5)	26 (100)	0.068
One sibling	0.51 (0.53)	48 (59.3)	33 (40.7)	81 (100)	
Two or more	0.61 (0.45)	14 (37.8)	23 (62.2)	37 (100)	
Relationship to child					
Father	0.42 (0.43)	35 (68.6)	16 (31.4)	51 (100)	0.010
Mother	0.59 (0.52)	50 (46.2)	43 (53.8)	93 (100)	
Monthly income					
Up to RM 5000	0.53 (0.57)	16 (51.6)	15 (48.4)	31 (100)	0.747
More than RM5000	0.53 (0.47)	62 (54.9)	51 (45.1)	113 (100)	
Family functioning: GF 12					
Good family functioning	0.46 (0.47)	69 (59.0)	48 (41.0)	117 (100)	0.016
Poor family functioning	0.82 (0.52)	9 (33.3)	18 (66.7)	27 (100)	
Dental caries status					
Caries free	0.33 (0.39)	53 (72.6)	20 (27.4)	73 (100)	< 0.001
Caries	0.73 (0.51)	25 (35.2)	46 (64.8)	71 (100)	

### Predictors of HRQOL

Table 5 shows the bivariate regression analysis with unadjusted and adjusted models. Unadjusted analysis was carried out to assess the association of the overall ECOHIS scores with socio-demographics, dental caries status and family functioning, significance was noted for two or more number of siblings (*P* = 0.067), relationship of the respondent to the child as mother (*P* = 0.011) and having dental caries (*P* ≤ 0.001). These significant variables were adjusted with each other up to three models based on significance (*P* = 0.200). First model was adjusted for sociodemographic variables of number of siblings and relationship to the child, both remained significant (*P* = 0.092 and *P* = 0.010 respectively). In the second model family functioning was adjusted with previous two variables and all three were significant: number of siblings (*P* = 0.050) and relationship to the child (*P* = 0.027) and family functioning (0.020). In the final third model, dental caries status was added which indicated that family functioning is no more significant (*P* = 0.917) whereas having two or more siblings (*P* = 0.013), relationship of the respondent to the child (*P* = 0.013) and dental caries status (*P* ≤ 0.001) were significantly associated with the HRQoL. The odds suggested; children with two or more siblings were more likely to have poor HRQoL (4.73), whose responses were filled by mothers were more likely to have poor HRQoL (2.86) and those with dental caries were more likely to have poor HRQoL (8.12) as compared to those with no caries.

**Table 5:** Unadjusted and adjusted odds of the association between ECOHIS scores and exploratory variables

Variables	Unadjusted			Model 1			Model 2			Model 3		
	OR	95% CI	*p* value	Adjusted OR	95% CI	*p* value	Adjusted OR	95% CI	*p* value	Adjusted OR	95% CI	*p* value
Demographic variables												
Age												
4 years	0.85	(0.38–1.87)	0.687									
5 years	1.41	(0.58–3.38)	0.439									
6 years	1.00											
Gender												
Male	1.27	(0.65–2.48)	0.474									
Female	1.00											
Siblings												
No siblings	1.00			1.00			1.00			1.00		
One sibling	1.10	(0.44–2.72)	0.837	0.96	(0.37–2.45)	0.938	1.01	(0.38–2.66)	0.975	0.82	(0.28–2.34)	0.712
Two or more	2.62	(0.93–7.38)	**0.067**	2.48	(0.86–7.17)	0.092	3.00	(1.00–9.02)	**0.050**	4.73	(1.38–16.23)	**0.013**
Relationship to child												
Father	1.00			1.00			1.00			1.00		
Mother	2.54	(1.24–5.21)	**0.011**	2.64	(1.26–5.54)	**0.010**	2.33	(1.09–4.97)	**0.027**	2.86	(1.24–6.59)	**0.013**
Monthly income												
Up to RM 5000	1.00											
More than RM5000	0.87	(0.39–1.94)	0.747									
Family functioning: GF 12												
Good FAD	1.00						1.00			1.00		
Poor FAD	2.87	(1.19–6.93)	**0.019**				3.02	(1.19–7.67)	**0.020**	1.05	(0.37–3.02)	0.917
Dental caries status												
Caries free	1.00									1.00		
Caries	4.87	(2.40–9.90)	**< 0.001**							8.12	(3.17–20.83)	**< 0.001**

Bold are statistically significant

Binary regression analysis: independent variables adjusted up to 3 Models based on significance (0.2)

## Discussion

This cross-sectional study focused on Chinese ethnicity, is the first study to observe family functioning as a mediating factor of disease-specific OHRQoL among pre-school children with childhood dental caries in Malaysia. Findings of this study indicates that children with two or more number of siblings, relationship with their respondent as mother and dental caries status are independently associated with health-related quality of life among 4 to 6-year-old pre-school Chinese children. Insight of these impacts will aid the clinicians and researchers to evaluate oral health needs, ascertain priorities of care and gauge the outcomes of various treatment options.

Overall good family functioning and OHRQoL were observed among the study sample. The reason could be the socioeconomic factors of the sample, that only included pre-school children from an international/private school setting reflecting of high socioeconomic background. More than three-fourth of the parents belonged to medium tier—M40 income group and contributes to 40% of the country's total income [24]. Chinese traditional culture is known for its strong and distinct family ethics, as part of this principled code of conduct in fostering children as their responsibilities, and in turn it ponders contentment and happiness.

Family functioning is also associated with children’s oral health. In contrast to previous findings [9, 35], this study found a significant association between family functioning and childhood dental caries. Almost all the children from poorer functioning families had dental decay as compared to children from normal functioning families. Significant findings were noted with parents relationship to child with family functioning, it indicates the possibility that better family functioning is reported by fathers that is reflective of dominant role of males in Chinese culture [36]. Expectations of mothers as compared to fathers, as they are more sensitive to family functioning. Females are more likely to disclose the emotional details about family functioning as compared to males [37].

Quality of life is impacted by oral health under physical, social and psychological domains [38]. Statistically significant association between family functioning reported by parents and assessment of their child's OHRQoL. This finding is consistent with previous studies that indicated an association between family functioning and HRQoL [39, 40].

The adverse impact of dental caries on children’s OHRQoL is also reported in this study and is supported by previously reported findings. These impacts include dentofacial pain, trouble chewing and sleeping that causes behavioural changes and compromises school performance [41–43].

In support to the previous studies, findings of this study confirms the relationship between the occurrence of dental caries and OHRQoL in children [14, 16, 44]. Several studies have verified the children’s parents responses for the OHRQoL [13–16], indicating that dental caries’ impact on children’s life is frequently related to the symptoms, limitations and psychological aspects. The finding of this study supports the previous findings with significance observed in all domains except in symptom domain. The lack of association between symptom domain and caries status could be explained by the levels of caries severity observed during clinical examination. Almost all the children in this study had low severity that could reflect on child’s perception of pain in symptoms domain. Dental caries is marked as slow progressing disease with relatively stable clinical signs (white or light brown spots). During this stage the symptoms are not perceived by lay individuals as an indicator of disease activity or latency. The symptoms are more recognizable as the disease progresses (brown spots with slight or moderate pain) [45].

Locker proposed that the link between oral disease and HRQoL outcomes is interceded by individual’s personal and environmental factors [46]. The findings of this study show three independent predictors for HRQoL. The first one, children having two or more siblings were more likely to have poor HRQoL. This finding is supported by resource dilution model (blake 1981), suggesting that a child’s development is affected by parental resource investment in terms of financial as well as time and attention provided to each child. This reflects on child’s educational outcomes, healthy lifestyle and overall wellbeing [47–49]. The second predictor to poor HRQoL in this study is the mothers’ relationship to the child as a respondent. Studies suggest that during caregiving mothers have a tendency to be more tired, emotionally stressed and upset as compared to fathers [50, 51]. It is also evident that females tend to share more emotional experience and can easily talk to others as compared to males [52]. The third predictor is dental caries, our study shows that children with dental caries are eight times more likely to have poor HRQoL as compared to those without caries. Previous evidence strongly supports this finding where dental caries is a significant predictor of HRQoL [14, 16, 53, 54]. The highly widespread dental ailment that affects nearly 60–90% of children is dental caries. This results in pain and functional limitation in children which in turns leads to poor HRQoL [55, 56]. Ungar explained the concept of resilience among children who face adversity, good family functioning can be conceptualized as a relational protective process that predict positive outcomes [57]. However in this study family functioning does not mediate in poor OHRQoL in the presence of dental caries.

Reliable and validated measures to assess OHRQoL and family functioning were used in this study, which contributes as one of the strengths. However, some probable limitations should be taken into consideration, that includes our sample being from one school setting and from one ethnic group that belonged to affluent social class thus indicating for the limited generalizability. Another concern could be of using self-report approaches that depend on parents’ perceptions and beliefs and they could be diverse from the actual behaviours [58]. Alternative possible limitation might be the 'Hawthorn effect' (parents’ reactivity to modify their behaviour in order to avoid embarrassment) [59]. Perception on family functioning may be varied based on which parent filled the questionnaire, this is accounted for study limitation. The cross-sectional nature of study design was one of the limitations associated with this research. Therefore, the results of this study are vital for clinicians and public health specialists in Malaysia intending to determine precise interventions to improve HRQoL. This study offers a basis for further longitudinal study studies to have more understanding towards the role of family functioning as a mediator between dental caries and HRQoL in the Malaysian context.

## Conclusion

Findings of this study indicates that caries status, number of siblings and relationship of the respondent to the child are independently associated with health-related quality of life. Health promoters should consider family functioning as an independent potential contributing factor of dental caries. Children with dental caries have a negative impact on HRQoL, this is an important indicator which can affect their growth, future self-esteem, and social life.

## Data Availability

All data are held by School of Dentistry, International Medical University under data storage guidelines.

## Abbreviations

OHRQoL: Oral health related quality of life
ECOHIS: Early Childhood Oral Health Impact Scale
FAD-GF12: Family functioning—12-item general functioning subscale

## References

[1] Holst D, Schuller AA, Aleksejuniene J, Eriksen HM (2001). Caries in populations: a theoretical, causal approach. Eur J Oral Sci.

[2] Kawashita Y, Kitamura M, Saito T (2011). Early childhood caries. Int J Dent.

[3] Narvey A, Shwart L (2007). Early childhood dental disease—what’s in a name?. J Can Dent Assoc.

[4] Harris R, Nicoll AD, Adair PM, Pine CM (2004). Risk factors for dental caries in young children: a systematic review of the literature. Community Dent Health.

[5] van Palenstein Helderman WH, Soe W, van’t Hof MA (2006). Risk factors of early childhood caries in a southeast asian population. J Dent Res.

[6] Schroth RJ, Halchuk S, Star L (2013). Prevalence and risk factors of caregiver reported severe early childhood caries in manitoba first nations children: results from the RHS Phase 2 (2008–2010). Int J Circumpolar Health.

[7] Duijster D, Verrips GHW, van Loveren C (2014). The role of family functioning in childhood dental caries. Community Dent Oral Epidemiol.

[8] Epstein NB, Ryan CE, Bishop DS, Miller IW, Keitner G. The McMaster model: a view of healthy family functioning. In: Normal family processes: growing diversity and complexity, 3rd edn. New York: The Guilford Press; 2003. p. 581–607.

[9] Hooley M, Skouteris H, Boganin C, Satur J, Kilpatrick N (2012). Parental influence and the development of dental caries in children aged 0–6 years: a systematic review of the literature. J Dent.

[10] Walsh F (2003). Family resilience: a framework for clinical practice. Fam Process.

[11] Wan Othman W, Abd Muttalib K, Mohamad A, Tan B-S, Zurina A, Che Salleh N, et al. The National Oral Health Survey of Preschool Children 2005 (NOHPS 2005) Oral Health Status and Treatment Needs. Kuala Lumpur; 2005.

[12] Health Information Management System, Malaysia. Oral Health Subsystem. Kuala Lumpur; 2012.

[13] de Andrade LHR, Buczynski AK, Raggio Luiz R, Castro GF, Pomarico Ribeiro de Souza I. Impacto de la salud oral en la calidad de vida de los niños pre-escolares: percepción de los responsables. Acta Odontológica Venez. 2011;49(4):15–6.

[14] Wong HM, McGrath CPJ, King NM, Lo ECM (2011). Oral health-related quality of life in hong kong preschool children. Caries Res.

[15] Kramer PF, Feldens CA, Helena Ferreira S, Bervian J, Rodrigues PH, Peres MA (2013). Exploring the impact of oral diseases and disorders on quality of life of preschool children. Community Dent Oral Epidemiol.

[16] Abanto J, Carvalho TS, Mendes FM, Wanderley MT, Bönecker M, Raggio DP (2011). Impact of oral diseases and disorders on oral health-related quality of life of preschool children. Community Dent Oral Epidemiol.

[17] Zubrick S, Australia. Department of Family and Community Services. AA, Silburn SR, Vimpani G. Indicators of social and family functioning. Canberra: Department of Family and Community Services; 2000.

[18] Wen LM, Simpson JM, Baur LA, Rissel C, Flood VM (2011). Family functioning and obesity risk behaviors: implications for early obesity intervention. Obesity.

[19] Renzaho AMN, de Silva-Sanigorski A (2014). The importance of family functioning, mental health and social and emotional well-being on child oral health. Child Care Health Dev.

[20] Tang C, Quinonez RB, Hallett K, Lee JY, Kenneth WJ (2005). Examining the association between parenting stress and the development of early childhood caries. Community Dent Oral Epidemiol.

[21] Bonanato K, Paiva SM, Pordeus IA, Ramos-Jorge ML, Barbabela D, Allison PJ (2009). Relationship between mothers’ sense of coherence and oral health status of preschool children. Caries Res.

[22] Lenčová E, Pikhart H, Broukal Z, Tsakos G (2008). Relationship between parental locus of control and caries experience in preschool children—cross-sectional survey. BMC Public Health.

[23] Lewison G, Kumar S, Wong C-Y, Roe P, Webber R (2016). The contribution of ethnic groups to Malaysian scientific output, 1982–2014, and the effects of the new economic policy. Scientometrics.

[24] Department of Statistics Malaysia Officail Portal. 2021. https://www.dosm.gov.my/v1/index.php?r=column/ctwoByCat&parent_id=119&menu_id=amVoWU54UTl0a21NWmdhMjFMMWcyZz09.

[25] WHO | Oral health surveys: basic methods, 5th edition. WHO. São Paulo, Brazil: World Health Organization; 2018. 125 p.

[26] Pahel BT, Rozier RG, Slade GD (2007). Parental perceptions of children’s oral health: the Early Childhood Oral Health Impact Scale (ECOHIS). Health Qual Life Outcomes.

[27] Hashim AN, Yusof ZYM, Esa R (2015). The Malay version of the Early Childhood Oral Health Impact Scale (Malay-ECOHIS)—assessing validity and reliability. Health Qual Life Outcomes.

[28] Epstein N, Baldwin L. The McMaster family assessment device. Marital Fam Ther. 1983.

[29] Mansfield AK, Keitner GI, Dealy J (2015). The family assessment device: an update. Fam Process.

[30] Shek DTL (2001). The general functioning scale of the family assessment device: does it work with chinese adolescents?. J Clin Psychol.

[31] Wo S, Lai P, Ong L, Low W, Wu D (2018). Factorial Validation of the Chinese General Functioning Subscale (Gf-12) Of The Family Assessment Device in Malaysia. J Heal Transl Med..

[32] World Health Organization. Oral Health Survey Basics Methods.2013. URL: http://www.who.int/oral_health/publicationspep_annex2formchildrentooth.pdf?.

[33] Goettems ML, Ardenghi TM, Romano AR, Demarco FF, Torriani DD (2011). Influence of maternal dental anxiety on oral health-related quality of life of preschool children. Qual Life Res.

[34] Altman D (1996). Practical statistics for medical research.

[35] Hallett K, O’Rourke P (2003). Social and behavioural determinants of early childhood caries. Aust Dent J.

[36] Hong CY, Baharudin R, Hossain Z (2012). Fathers’ parenting styles in Chinese families in urban Malaysia. Pertanika J.

[37] Buck R (1977). Nonverbal communication of affect in preschool children: relationships with personality and skin conductance. J Pers Soc Psychol.

[38] Tesch FC, de Oliveira BH, Leão A (2008). Equivalência semântica da versão em português do instrumento Early Childhood Oral Health Impact Scale. Cad Saude Publica.

[39] Curt LaFrance Jr. W, Alosco ML, Davis JD, Tremont G, Ryan CE, Keitner GI, et al. Impact of family functioning on quality of life in patients with psychogenic nonepileptic seizures versus epilepsy. Epilepsia. 2011;52(2):no-no. 10.1111/j.1528-1167.2010.02765.x 21299547

[40] Speechley KN, Ferro MA, Camfield CS, Huang W, Levin SD, Lou SM (2012). Quality of life in children with new-onset epilepsy: a 2-year prospective cohort study. Neurology.

[41] Feitosa S, Colares V, Pinkham J (2005). The psychosocial effects of severe caries in 4-year-old children in Recife, Pernambuco. Brazil Cad Saude Publica.

[42] Versloot J, Veerkamp JSJ, Hoogstraten J (2006). Dental Discomfort Questionnaire: assessment of dental discomfort and/or pain in very young children. Community Dent Oral Epidemiol.

[43] Clarke M, Locker D, Berall G, Pencharz P, Kenny DJ, Judd P (2006). Malnourishment in a population of young children with severe early childhood caries. Pediatr Dent.

[44] Goettems ML, Ardenghi TM, Romano AR, Demarco FF, Torriani DD (2011). Influence of maternal dental anxiety on oral health–related quality of life of preschool children. Qual Life Res.

[45] Mafla AC, Villalobos-Galvis FH, Heft MW (2018). Illness perceptions amongst individuals with dental caries. Community Dent Health.

[46] Locker D (2007). Disparities in oral health-related quality of life in a population of Canadian children. Community Dent Oral Epidemiol.

[47] Blake J (1981). Family size and the quality of children. Demography.

[48] Downey DB (1995). When bigger is not better: family size, parental resources, and children’s educational performance. Am Sociol Rev.

[49] Gibbs BG, Workman J, Downey DB (2016). The (conditional) resource dilution model: state- and community-level modifications. Demography.

[50] McDonnell C, Luke N (2019). Short happy moms, happier dads: gendered caregiving and parents’ affect. J Family Issues.

[51] Musick K, Meier A, Flood S (2016). How parents fare: Mothers’ and fathers’ subjective well-being in time with children. Am Sociol Rev.

[52] Brody LR, Hall JA, Lewis M, Haviland-Jones JM, Feldman-Barrett L (2008). Gender and emotion in context. Handbook of emotions.

[53] Chukwumah NM, Folayan MO, Oziegbe EO, Umweni AA (2016). Impact of dental caries and its treatment on the quality of life of 12-to 15-year-old adolescents in Benin. Nigeria Int J Paediatr Dent.

[54] Masumo R, Bardsen A, Mashoto K, Astrom AN (2012). Prevalence and socio-behavioral influence of early childhood caries, ECC, and feeding habits among 6–36 months old children in Uganda and Tanzania. BMC Oral Health.

[55] Mashoto KO, Astrom AN, David J, Masalu JR (2009). Dental pain, oral impacts and perceived need for dental treatment in Tanzanian school students: a cross-sectional study. Health Qual Life Outcomes.

[56] Petersen PE, Bourgeois D, Ogawa H, Estupinan-Day S, Ndiaye C (2005). The global burden of oral diseases and risks to oral health. Bull World Health Organ.

[57] Ungar M (2006). Resilience across cultures. Br J Soc Work.

[58] Schwarz JC, Barton-Henry ML, Pruzinsky T (1985). Assessing child-rearing behaviors: a comparison of ratings made by mother, father, child, and sibling on the CRPBI. Child Dev.

[59] McCarney R, Warner J, Iliffe S, van Haselen R, Griffin M, Fisher P (2007). The hawthorne effect: a randomised, controlled trial. BMC Med Res Methodol.

